# Asparagus Officinalis as a Remedy for Hydrophobia

**Published:** 1853-10

**Authors:** 


*Asparagus Officinalis as a Remedy for Hydrophobia.*—Dr. Chairétes, of Athens, reports in the London Lancet, 27th August, 1853, some cases which he deems illustrative of the curative powers of Asparagus in hydrophobia.

